# Whole Exome Sequencing Reveals Genetic Variants in HLA Class II Genes Associated With Transplant-free Survival of Indeterminate Acute Liver Failure

**DOI:** 10.14309/ctg.0000000000000502

**Published:** 2022-06-02

**Authors:** Tsung-Jen Liao, Bohu Pan, Huixiao Hong, Paul Hayashi, Jody A. Rule, Daniel Ganger, William M. Lee, Jorge Rakela, Minjun Chen

**Affiliations:** 1 Division of Bioinformatics and Biostatistics, U.S. Food and Drug Administration (FDA) National Center for Toxicological Research, Jefferson, Arkansas, USA;; 2 Division of Hepatology and Nutrition, Office of New Drugs, FDA Center for Drug Evaluation and Research, Silver Spring, Maryland, USA;; 3 Division of Gastroenterology and Hepatology, University of Texas Southwestern, Dallas, Texas, USA;; 4 Division of Gastroenterology and Hepatology, Northwestern University, Chicago, Illinois, USA;; 5 Division of Gastroenterology and Hepatology, Mayo Clinic, Phoenix, Arizona, USA.

## Abstract

**INTRODUCTION:**

Indeterminate acute liver failure (IND-ALF) is a rare clinical syndrome with a high mortality rate. Lacking a known etiology makes rapid evaluation and treatment difficult, with liver transplantation often considered as the only therapeutic option. Our aim was to identify genetic variants from whole exome sequencing data that might be associated with IND-ALF clinical outcomes.

**METHODS:**

Bioinformatics analysis was performed on whole exome sequencing data for 22 patients with IND-ALF. A 2-tier approach was used to identify significant single-nucleotide polymorphisms (SNPs) associated with IND-ALF clinical outcomes. Tier 1 identified the SNPs with a higher relative risk in the IND-ALF population compared with those identified in control populations. Tier 2 determined the SNPs connected to transplant-free survival and associated with model for end-stage liver disease serum sodium and Acute Liver Failure Study Group prognostic scores.

**RESULTS:**

Thirty-one SNPs were found associated with a higher relative risk in the IND-ALF population compared with those in controls, of which 11 belong to the human leukocyte antigen (HLA) class II genes but none for the class I. Further analysis showed that 5 SNPs: rs796202376, rs139189937, and rs113473719 of HLA-DRB5; rs9272712 of HLA-DQA1; and rs747397929 of IDO1 were associated with a higher probability of IND-ALF transplant-free survival. Using 3 selected SNPs, a model for the polygenic risk score was developed to predict IND-ALF prognoses, which are comparable with those by model for end-stage liver disease serum sodium and Acute Liver Failure Study Group prognostic scores.

**DISCUSSION:**

Certain gene variants in HLA-DRB5, HLA-DQA1, and IDO1 were found associated with IND-ALF transplant-free survival. Once validated, these identified SNPs may help elucidate the mechanism of IND-ALF and assist in its diagnosis and management.

## INTRODUCTION

Acute liver failure (ALF) is an uncommon clinical condition, which can lead to rapid deterioration of liver function in patients without preexisting liver disease. ALF is fatal and characterized by a high mortality rate, which ranges from 40% to 85% without liver transplantation (1,2). Its causes include acetaminophen overdose (3), drug-induced liver injury (4), various hepatitis viruses (5), autoimmune diseases (6), and others (7). In the United States, approximately 2,000–3,000 cases per year are diagnosed as ALF (8,9); only approximately 5% of the cases were considered indeterminate ALF (IND-ALF) (10–14). Liver transplantation (LT) is often viewed as the only therapeutic treatment of IND-ALF (15,16). Therefore, identifying prognostic factors to predict transplant-free survival is of particular importance in improving its diagnosis and treatment.

Prognostic factors associated with transplant-free ALF survival include coma grade, etiology, vasopressor usage, bilirubin, and international normalized ratio (17). Complications such as severe coagulopathy and higher coma grade might identify those patients who would die without LT (18,19). A recent investigation also indicated that coagulation factor V can be used to improve prediction of transplant-free ALF survival (20). Although multiple risk factors for ALF associated with clinical and laboratory data have been reported, genetic factors for predicting transplant-free survival have not been fully explored.

In our previous study, the potential genetic variants associated with IND-ALF were uncovered by analyzing whole exome sequencing (WES) data obtained from the Acute Liver Failure Study Group (ALFSG) (21). The ALFSG identified and placed in a registry 2,718 patients with ALF in the United States from 1998 to 2016; 150 patients were deemed to have IND-ALF (11). DNA samples were collected from 26 of these patients. By comparing all the genetic variants with the allele frequency of the control group (AF^control^) greater than 1% in gnomAD v2, several single-nucleotide polymorphisms (SNPs) were identified (21). The most common SNP, rs4940595 of the SERPINB11 gene, was found in 23 of the 26 patients with IND-ALF. The SNPs rs1800754 of the CYP2D7 gene and rs1135840 of the CYP2D6 gene seem to have survival-protective effects because of asymmetric distribution between those patients who spontaneously survived (75%) versus those who died or underwent liver transplantation (30.5% and 25%, respectively). Recently, rare and low-frequency genetic variants were reported to contribute to missing heritability and could be prevalent in the protein-coding region (22–24). We therefore used the latest version of the control database, gnomAD v3, which contains 5-fold more genome samples than gnomAD v2. Our analysis of the WES data focused on genetic variants that might be associated with transplant-free IND-ALF survival.

Our work in this pilot study aimed to investigate the hypothesis of the association of minor/rare gene variations with the clinical outcome of IND-ALF. With more clinical data elucidated, the ALFSG causality committee identified a specific etiology for 4 of the 26 patients with IND-ALF previously reported (21). In this study, we analyzed the WES data for the remaining 22 patients with IND-ALF and revealed the high allele frequency of SNPs in human leukocyte antigen (HLA) genes. HLA modulates immune response and has been associated with liver diseases (25). By using the selected significant SNPs, a polygenic risk score (PRS) predicted the clinical outcome for patients with IND-ALF, with the results comparable with those found by the end-stage liver disease with serum sodium (MELD-Na) score and ALFSG prognostic score. These significant SNPs may be used as prognostic genetic factors for predicting transplant-free survival in patients with IND-ALF.

## RESULTS

### SNPs of HLA genes prevail in the 22 patients with IND-ALF

**Figure 1.**
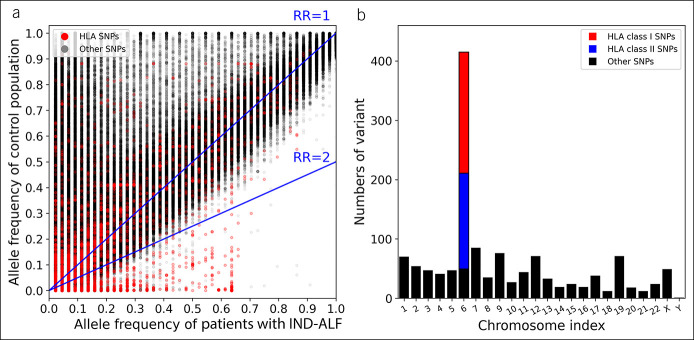
Potential genetic variants associated with indeterminate acute liver failure (IND-ALF) were identified. (**a**) A total of 478,561 single-nucleotide polymorphisms (SNPs) with genotype quality (GQ) ≥20, depth of coverage (DP) ≥10, higher impact, and predictive transcript biotype in protein coding were shown the comparison of allele frequency obtained from the gnomAD database (control population) vs the 22 patients with IND-ALF. The human leukocyte antigen (HLA) SNPs were colored red, and other SNPs were colored grey. The ratio of patients to control population indicates the relative risk (RR, blue). (**b**) A total of 1,332 SNPs with RR > 2 and AFIND-ALF >0.2 were selected. Chromosome 6 contains the largest number of variants: 204 SNPs belong to HLA class I (red) and 161 SNPs are HLA class II (blue). SNPs not associated with HLA genes were colored black.

The preprocessed genetic variants were first filtered by genotype quality, putative impact, and transcript biotype, yielding 478,561 variants (Figure 1a). In this study, we focused on variants with a higher relative risk (RR) and allele frequency (AF) of the patients with IND-ALF (AF^IND-ALF^) as compared with the AF of controls (AF^control^). The control population combined the non-Finnish European (n = 34,079), African American (n = 20,744), and Eastern Asian (n = 2,604) populations from gnomAD. For our study population with 22 IND-ALF patients, a generic variant with a threshold of AF^IND-ALF^ > 0.2 compared with the allele frequency AF^control^ < 0.1 would perform at the statistical power >0.8 with type I error 0.05. Therefore, we used RR (AF^IND-ALF^/AF^control^) > 2 and AF^IND-ALF^ > 0.2 as the thresholds, and a total of 1,332 variants were selected for further analysis. We found that 365 of these selected variants were associated with HLA genes, including 204 variants of HLA class I genes (A, B, and C) and 161 of HLA class II genes (DP, DQ, and DR) (Figure 1b). We did not consider the alleles with high frequency in the control population but low frequency in the IND-ALF because we only have 22 patients with ALF, which cannot provide robust estimation of the allele with low frequency (e.g., < 0.1).

Notably, SNPs of HLA genes prevailed among the high-frequency alleles in our 22 patients with IND-ALF. For example, there were a total of 109 variants with AF^IND-ALF^ > 0.5, with 96 belonging to HLA class I (n = 87) and class II (n = 9) genes (see Table S1, Supplementary Digital Content 1, http://links.lww.com/CTG/A826). HLA-C contained the largest number of SNPs (n = 62), followed by A (n = 17), B (n = 8), DQ (n = 8), and DR (n = 1).

### SNPs of HLA class II genes are associated with transplant-free survival

The genetic factors for transplant-free survival were uncovered by examining the selected 1,332 SNPs through the Fisher exact test for the liver transplant/death vs survival groups, and only 31 SNPs were found to be statistically significant (*P*-value ≤ 0.05) (Table 1). Of the 31 SNPs, 11 were related to the HLA class II genes, but none belonged to the HLA class I genes. Of the 11 SNPs, 7 were found in DRB5 and 4 in DQA1, respectively. The 7 SNPs in HLA-DRB5—rs796202376, rs139189937, rs372470599, rs796546531, rs113473719, rs75563047, and rs151002802—had mutant outcomes in the 3' untranslated region (UTR), of which the RR ranges from 6 to 23. By contrast, for the 4 SNPs in HLA-DQA1, rs1129808 and rs1142334, resulted in mutations Y103S and M89I with RR > 100. The remaining SNPs in HLA-DQA1, rs9272712 and rs9272722, had mutations in the intron region.

**Table 1.** Genetic variants with significant association with LT/death vs spontaneous survival groups

Gene	SNP ID	Position	Mutation	Outcome	No. of patients	RR	Fisher *P*
HLA-related							
HLA-DRB5	rs796202376	chr6:32517663	A→G	3' UTR	7	23.42	2.5 × 10^−4^
	rs139189937	chr6:32517674	A→G	3' UTR	7	16.74	2.5 × 10^−4^
	rs372470599	chr6:32517644	T→G	3' UTR	6	16.53	2.1 × 10^−5^
	rs796546531	chr6:32517645	C→T	3' UTR	6	15.64	1.2 × 10^−4^
	rs113473719	chr6:32517690	A→C	3' UTR	7	13.79	2.5 × 10^−4^
	rs75563047	chr6:32517715	C→T	3' UTR	6	9.00	2.1 × 10^−5^
	rs151002802	chr6:32517633	G→T	3' UTR	5	6.73	5.5 × 10^−4^
HLA-DQA1	rs1129808	chr6:32641535	A→C	Y103S	10	140.13	4.2 × 10^−2^
	rs1142334	chr6:32641494	G→C	M89I	7	104.70	3.2 × 10^−2^
	rs9272712	chr6:32641564	C→T	Intron	10	98.58	4.9 × 10^−2^
	rs9272722	chr6:32641627	C→T	Intron	10	12.77	4.9 × 10^−2^
Others							
CDK11A	rs77869096	chr1:1713611	C→T	Intron	16	3.12	4.3 × 10^−3^
RAVER2	rs60101975	chr1:64833091	G→GTGTTGTT	Noncoding	6	3.10	4.0 × 10^−2^
PHGDH	rs2236400	chr1:119744318	G→A	Downstream	8	2.70	4.0 × 10^−2^
PHGDH	rs11487674	chr1:119744327	T→C	Downstream	8	2.62	4.0 × 10^−2^
GYPE	rs4091835	chr4:143903193	T→G	Intron	17	4.65	4.4 × 10^−2^
MTRF1L	rs201122580	chr6:152988208	C→T	—	10	170.72	8.2 × 10^−3^
MMS22L	rs3822909	chr6:97145067	A→C	Noncoding	6	2.37	4.0 × 10^−2^
IFRD1	rs7798142	chr7:112458216	T→C	Intron	12	2.01	4.2 × 10^−2^
GPR37	rs2239532	chr7:124765622	A→C	5’ UTR	16	2.03	1.0 × 10^−2^
COL28A1	rs10486176	chr7:7506060	G→C	T327S	9	2.36	1.2 × 10^−2^
IDO1	rs747397929	chr8:39918767	AAAC→A	Intron	7	8.68	1.9 × 10^−2^
MIR3689A	rs777254416	chr9:134849445	A→G	Downstream	5	9.09	1.8 × 10^−2^
PRSS3	rs151192741	chr9:33797863	A→G	K79E	11	5.23	8.8 × 10^−3^
PRSS3	rs1052839412	chr9:33797933	A→T	D102V	14	4.40	1.9 × 10^−2^
PRSS3	rs1331110514	chr9:33797941	G→A	D105N	13	5.10	3.5 × 10^−2^
BTG4	rs5794752	chr11:111496892	G→GAAA	Intron	9	2.72	3.2 × 10^−2^
PPFIA2	rs748481200	chr12:81268124	T→TTTTC	Intron	11	10.06	4.9 × 10^−2^
RFC3	rs1805375	chr13:33835114	C→G	Intron	9	2.11	2.1 × 10^−2^
FAM169B	rs58790202	chr15:98437681	T→TG	3' UTR	8	3.28	3.2 × 10^−2^
SAFB	rs148831822	chr19:5667193	AC→A	Intron	8	3.68	4.0 × 10^−2^

RR, relative risk (AF^IND-ALF^/AF^control^), and AF^IND-ALF^ represents allele frequency of the 22 patients with IND-ALF.

Fisher *P*: *P*-value of the Fisher exact test.

Dash (−): unreported outcome.

In addition to HLA, 20 genetic variants found in 17 genes had a higher frequency in the IND-ALF population. The rs77869096 of CDK11A had a high AF in the patients (AF^IND-ALF^ = 0.73) while the influence was in the intron region. The rs201122580 of MTRF1L had a very high RR (>170), and the consequence was unknown. Three SNPs rs151192741, rs1052839412, and rs1331110514 of PRSS3 resulted in the mutations K79E, D102V, and D105N, respectively, on the serine protease 3. In addition, rs10486176 of COL28A1 led to the mutation T327S on the collagen, type XXVIII, and alpha-1 chain. These variants were missense mutations without associations with existing diseases in literature reports.

### SNPs of HLA-DRB5, HLA-DQA1, and IDO1 are associated with the prognosis of IND-ALF

To reduce potential false-positives among the genetic markers, the Student *t* test was performed using MELD-Na scores and ALFSG indexes to further filter the selected SNPs. Five SNPs with *P*-value ≤ 0.05 for both MELD-Na and ALFSG were identified, including rs796202376, rs139189937, and rs113473719 in HLA-DRB5; rs9272712 in HLA-DQA1; and rs747397929 in IDO1 (Table 2). All 5 *t* test-significant SNPs had OR<1, suggesting that their presence was associated with increasing transplant-free survival possibility.

**Table 2.** Two-sample *t* test examines genetic variants for MELD-Na and ALFSG

Gene	SNP ID	LT/death vs survival	MELD-Na	ALFSG
		OR	2-sample *t* test *P*-value	
HLA-related				
HLA-DRB5	rs796202376	0.06	0.05^a^	3.9 × 10^−3^^a^
	rs139189937	0.06	0.05^a^	3.9 × 10^−3^^a^
	rs372470599	0.03	0.10	0.01^a^
	rs796546531	0.04	0.10	0.01^a^
	rs113473719	0.06	0.05^a^	3.9 × 10^−3^^a^
	rs75563047	0.03	0.10	0.01^a^
	rs151002802	0.05	0.26	0.02^a^
HLA-DQA1	rs1129808	0.23	0.06	0.03^a^
	rs1142334	0.20	0.22	0.09
	rs9272712	0.23	0.01^a^	0.01^a^
	rs9272722	0.23	0.06	0.03^a^
Others				
CDK11A	rs77869096	8.67	0.35	0.48
RAVER2	rs60101975	—	0.07	0.02^a^
PHGDH	rs2236400	—	0.15	0.05
PHGDH	rs11487674	—	0.15	0.05
GYPE	rs4091835	6.00	0.17	0.09
MTRF1L	rs201122580	9.00	0.22	0.44
MMS22L	rs3822909	—	0.46	0.44
IFRD1	rs7798142	0.23	0.09	0.06
GPR37	rs2239532	7.85	0.40	0.16
COL28A1	rs10486176	0.15	0.06	0.14
IDO1	rs747397929	0.15	0.01^a^	0.01^a^
MIR3689A	rs777254416	—	0.24	0.09
PRSS3	rs151192741	—	0.24	0.40
PRSS3	rs1052839412	9.94	0.15	0.26
PRSS3	rs1331110514	8.67	0.32	0.20
BTG4	rs5794752	0.20	0.17	0.47
PPFIA2	rs748481200	5.25	0.46	0.40
RFC3	rs1805375	0.15	0.29	0.10
FAM169B	rs58790202	0.20	0.11	0.16
SAFB	rs148831822	—	0.27	0.41

Unavailable odds ratios due to zero denominator are denoted by dash (—).

a *P* value ≤ 0.05.

**Figure 2.**
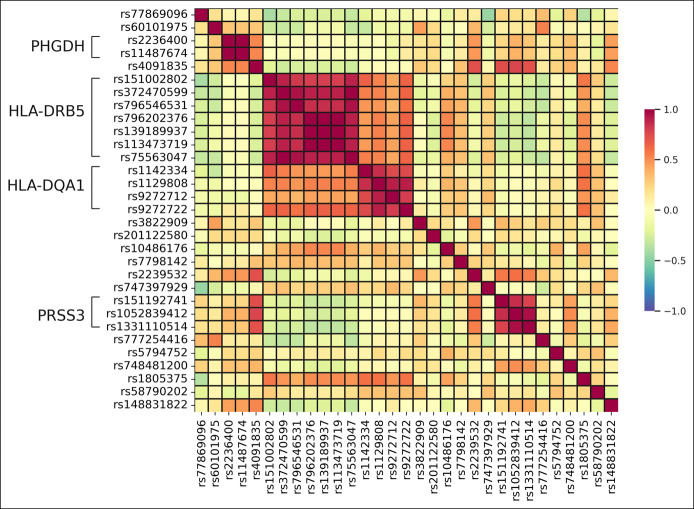
Correlations for the 31 genetic variants were examined using linkage disequilibrium. The correlation coefficient (r) ranges from −1 (blue) to 1 (red). Variants within the same gene were indicated.

Variants in nearby genes often show high correlation of allele frequency in populations. Here, SNPs with identical AF^IND-ALF^, odds ratio, and *t* test *P*-values were observed. Nonrandom association of alleles was estimated by the correlation calculation based on linkage disequilibrium. The 31 variants were examined, and variants in the same gene showed high positive correlation coefficients (*r* > 0.8) (Figure 2). SNPs located in HLA-DRB5 and HLA-DQA1 also showed moderate correlation (*r* > 0.5). Noticeably, the 3 SNPs rs796202376, rs139189937, and rs113473719 in HLA-DRB5 had correlation coefficient *r* = 1, which indicated that they had identical allele distribution in our patients with IND-ALF. Therefore, to prevent duplicate contributions from the 3 identical SNPs, we only kept rs796202376 for further analysis.

### PRS based on SNPs from HLA-DRB5, HLA-DQA1, and IDO1 identified 5 IND-ALF cases with low/medium risk

**Figure 3.**
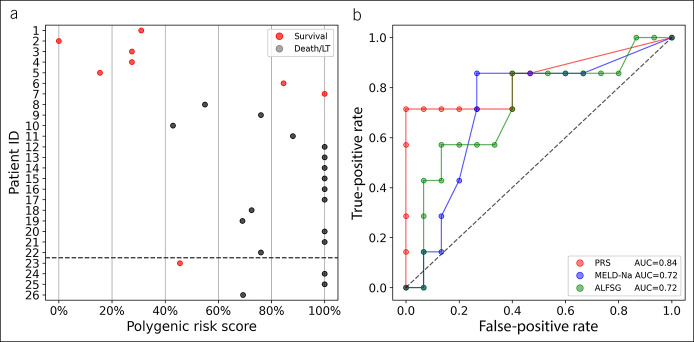
The polygenic risk score (PRS) predicts the risk for the 22 patients with indeterminate acute liver failure (IND-ALF) and compares them with the MELD-Na scores and Acute Liver Failure Study Group (ALFSG) indexes. (**a**) The predicted risk scaled from 0% (lowest risk) to 100% (highest risk). The patients who survived spontaneously were colored red, and patients who died or underwent liver transplantation (LT) were colored grey. The dashed line separates the patients with IND-ALF (1–22) and patients with identified etiologies (23–26). (**b**) The receiver operating characteristic (ROC) curve shows the true-positive rate (TPR) against the false-positive rate (FPR) at different classification thresholds. The areas under the curve (AUCs) of PRS (red), MELD-Na (blue), and ALFSG (green) are 0.84, 0.72, and 0.72, respectively.

A polygenic risk score (PRS) was constructed using the genetic variants passing 2-tier selections to gauge the patients' risks for IND-ALF. Three statistically significant SNPs—rs796202376 of HLA-DRB5, rs9272712 of HLA-DQA1, and rs747397929 of IDO1—were selected for assessment. The calculated PRS for 22 patients with IND-ALF showed the low/middle scores for 5 patients who survived spontaneously and middle/high scores for 15 dead/LT patients (Figure 3a). While 2 spontaneous survivors (patients 6 and 7) had high predicted risk scores (PRS>80%), no patients with low PRSs had IND-ALF that developed into LT or death.

In addition, the receiver operating characteristic curve was performed and applied to our PRS, MELD-Na score, and ALFSG prognostic score (Figure 3b). The PRS yielded an area under ROC curve (AUC) of 0.84, as compared with AUCs of 0.72 generated by the MELD-Na score and ALFSG prognostic score. This suggested that our PRS performed comparably with the MELD-Na score and ALFSG prognostic score in predicting prognoses for these patients with IND-ALF. We also constructed a PRS model by using 31 SNPs in Table 1, and it got a similar classification effect but with a higher AUC (see Figure S1, Supplementary Digital Content 1, http://links.lww.com/CTG/A826). We also combined the PRS with MELD-Na and/or ALFSG, but it would not improve the prediction of transplant-free survival. By taking the average risk scores for PRS/MELD-Na, PRS/ALFSG, and PRS/MELD-Na/ALFSG, the AUC values are similar (see Figure S2–S4, Supplementary Digital Content 1, http://links.lww.com/CTG/A826).

The PRS was validated using the 4 ALF patients with redefined etiologies (Figure 3a). Patient 23 had a lower/middle risk score and survived. By contrast, patients 24, 25, and 26 had middle/high risk scores and ultimately underwent LT or died. Overall, our calculated PRS performed an accurate evaluation of the prognoses of these 4 patients.

## DISCUSSION

The pathogenesis of IND-ALF is not well understood; outcomes are generally poor among this patient group. Here, we used a 2-tier approach to analyze WES data for 22 patients with IND-ALF and identified molecular markers among these low-frequency genetic variants. In tier 1, the potential variants with higher RR (>2) and AF^IND-ALF^ (>0.2) were considered significant and were kept. In tier 2, the variants associated with severe clinical outcomes (Fisher exact test, *P*-value ≤ 0.05) were used, and of those, only the variants that showed statistical significance (*t* test, *P*-value ≤ 0.05) for both MELD-Na and ALFSG prognostic scores were selected. After excluding those with identical allele distribution, 3 SNPs, including 2 HLA alleles, were considered the genetic risk factors. We developed a PRS model based on these selected SNPs, and the PRS's ability to predict clinical outcomes was comparable with that of the MELD-Na and ALFSG prognostic scores in our IND-ALF population.

However, the small study population might limit our statistical analysis for identifying the genetic variants. To reach the statistical power of >0.8 for each selected SNP, we chose the SNPs with AF^IND-ALF^>0.2 for our patients with IND-ALF (n = 22) and AF^control^<0.1 for the control groups (n = 57,427); thus, we used RR > 2. SNPs with AF^control^>0.1 were not included. It is still probable that some potential genetic variants are hidden in the higher allele frequency of the control population. Retrieving reliable SNPs with higher AF^control^ requires a larger study population of IND-ALF cases. Notably, we found that SNPs of HLA genes prevailed, especially among the high-frequency alleles in our 22 patients with IND-ALF, suggesting an association between HLA and IND-ALF.

Mutant HLA was often implicated in various autoimmune diseases (26). Various HLA alleles have been found to be associated with liver disease and liver toxicity caused by chemical drugs, as well as herbal and dietary supplements (27–29). HLA-DR variants have also been implicated in causing increased susceptibility to hepatitis B virus-related ALF (30), and the reduced expression of monocyte HLA-DR was observed in acetaminophen-induced ALF (31). Furthermore, HLA alleles were reported as prognostic factors for disease-free survival. For example, HLA class I allele HLA-B27/57 played a protective role against human immunodeficiency virus (HIV) and hepatitis C virus infections (32); HLA class II allele HLA-DRB*13 was associated with better survival for patients with non-small-cell lung cancer after receiving chemotherapy (33). In this study, we uncovered 5 potential genetic prognostic factors that might be associated with transplant-free survival in patients with IND-ALF. Nonetheless, none of these selected SNPs reflected the expression of the phenotype; HLA-DRB5 rs796202376, rs139189937, and rs113473719 influenced the 3' UTR while HLA-DQA1 rs9272712 and IDO1 rs747397929 affected the intron region.

HLA-DRB5 rs796202376, rs139189937, and rs113473719 had the largest contributions to PRS predictability with AF^IND-ALF^ = 0.32 and OR = 0.06. The mutant effects of 3' UTRs in HLA-DRB5 were undefined. It has been recognized that 3' UTRs can mediate messenger ribonucleic acid (mRNA) stability, translation, and localization (34,35). They may also associate with RNA-binding proteins and transmit genetic information, changing protein functions and features (36). In addition, HLA 3' UTRs can be the target of miRNA (37). Recent studies showed the binding of miRNA to 3' UTRs of HLA-B and HLA-G regulated the gene expression (38,39). The mutations on the 3' UTRs may affect the binding of miRNA. For example, the HLA-DRB5 SNP rs139189937 (A→G) led to a gain of miRNA hsa-miR-1287-5p and a loss of hsa-miR-873-5p targeting binding (40). The change of the binding to miRNA may regulate the expression of HLA-DRB5.

HLA-DQA1 rs9272712 with an AF^IND-ALF^ of 0.34 and an OR of 0.23 also contributed to the PRS prediction. The rs9272712 locates in the intron region, of which a function has rarely been reported. However, intron can reduce genetic instability (41), enhance the recruitment of RNA polymerase to the site of transcription, and increase the gene expression level (42,43). Although research on the introns of HLA lags far behind, a recent study suggested that HLA-DQA1 alleles were associated with protection from hepatitis B virus infection (44).

IDO1 rs747397929 has the smallest weight in the PRS compared with that of 2 other genetic variants. IDO1 represented indoleamine 2,3-dioxygenase 1 and was well characterized as a mediator of tumor immune evasion (45). Like rs9272712, rs747397929 also locates in the intron region with an unclear function. Noticeably, inhibition of IDO1 can induce immune-mediated liver injury in mice (46). Therefore, it is speculated that IDO1 might be involved in the regulation of the immune system and might favorably affect the clinical course of IND-ALF.

Thus, patients with IND-ALF with SNPs rs796202376, rs139189937, rs113473719, rs9272712, and rs747397929 may have better clinical outcomes with IND-ALF. In our data set, patients 6 and 7 were reported as spontaneous survivors because they were alive 21 days after their enrollment in the ALFSG study, but they were predicted as high-risk, with PRSs of 80% and 100%, respectively. After reviewing their medical records, we found that patient 6 was not sick enough for liver transplantation and survived. However, patient 7 did not undergo liver transplantation because of seizures and irreversible brain injury and was discharged 65 days after enrollment with persistent abnormal liver injury tests and without recovering consciousness; this patient subsequently died at home. By contrast, patients 8 and 10 had medium PRS and liver biopsies showing submassive necrosis and diffuse necrosis, respectively. Both recovered spontaneously without liver transplantation.

In summary, we investigated the WES data of 22 patients with IND-ALF. Statistical analysis identified 31 genetic variants, 11 of which were related to HLA genes. SNPs rs796202376, rs139189937, and rs113473719 in HLA-DRB5; rs9272712 in HLA-DQA1; and rs747397929 in IDO1 were significantly associated with better survival. Variants rs796202376, rs139189937, and rs113473719 had identical allele distribution in these patients. Using the selected SNPs, the PRS prediction performed similarly to that of MELD-Na scores and ALFSG prognostic scores. Because all 5 SNPs showed association with an increasing survival rate, these genetic variants might regulate the corresponding gene expression and protect patients with IND-ALF from death. Although these potential SNPs require further validation in a larger patient population, they may help differentiate those patients who would spontaneously recover from those who may require liver transplantation.

## MATERIAL AND METHODS

### Study population

The 22 patients satisfying the criteria of IND-ALF were assigned ID numbers 1 to 22, and 4 additional ALF patients with identified etiologies were numbered 23 to 26. Demographic, laboratory, and clinical data are summarized in Table 3. Clinical outcomes were determined 21 days after enrollment. Among the IND-ALF patients 1–22, 7 patients spontaneously recovered, 10 received liver transplantation, and 5 died. For patients 23–26, 1 spontaneously recovered, 2 received liver transplantation, and 1 died. All patients with liver transplants were alive after 21 days. The self-identified ethnicity shows 16 Whites, 5 African Americans, and 1 Eastern Asian for patients with IND-ALF and 4 Whites for the ALF patients with identified etiologies. Male and female patients were equally distributed. Liver enzymes, including total bilirubin, aspartate aminotransferase, alanine aminotransferase, alkaline phosphatase, and creatinine, were measured when the patients were enrolled. MELD-Na and ALFSG scores were also assessed.

**Table 3.** Clinical characteristics of 22 IND-ALF and 4 ALF patients

Patient ID	Age (yr)	Sex	Ethnicity	Outcome	TBil (mg/dL)	AST (unit/L)	ALT (unit/L)	ALP (unit/L)	INR	Coma	Creatinine (mg/dL)	MELD-Na	ALFSG
Indeterminate etiology													
1	24	M	White	S	3.3	1945	1760	223	1.8	1	1.3	28	74%
2	87	M	White	S	4.3	1,000	500	269	2.3	2	2.9	31	63%
3	53	F	White	S	14.2	662	742	132	1.9	2	0.9	24	49%
4	19	M	Eastern Asian	S	33.3	406	1,178	274	1.6	2	0.7	30	40%
5	36	M	White	S	27.2	724	571	128	2.4	1	0.8	29	30%
6	38	M	White	S	5.9	3,850	1997	116	2.8	3	1.8	30	29%
7	30	F	African American	S	24.7	4,754	7,012	126	5.3	3	2.6	40	6%
8	19	M	White	LT	29.1	1996	2,600	155	8.8	1	1.6	40	42%
9	29	F	White	LT	8.2	96	207	96	1.8	4	0.6	22	37%
10	52	F	White	LT	15.9	320	322	175	2.6	2	1.1	29	36%
11	37	F	White	LT	9.2	669	386	121	4.3	1	1.5	36	30%
12	22	F	White	LT	29.8	331	163	306	2.6	1	0.8	30	27%
13	30	M	White	LT	36.5	875	1,412	121	1.3	4	1.5	27	24%
14	30	M	African American	LT	26.8	329	262	67	4.2	2	3.8	40	17%
15	26	F	White	LT	19.6	492	460	238	5.7	2	0.8	37	15%
16	46	F	White	LT	26.7	1922	1,476	260	3.2	4	1.0	32	10%
17	47	F	White	LT	22.7	2,820	5,670	150	16.4	2	0.4	40	4%
18	47	F	African American	D	3.7	51	27	85	5.6	2	1.1	32	84%
19	62	M	African American	D	23.6	—	1829	72	2.4	2	1.5	32	33%
20	39	M	White	D	20.1	648	551	795	1.6	4	6.0	36	27%
21	46	M	White	D	25.5	180	194	245	2.0	4	10.1	40	6%
22	23	F	African American	D	17.6	1,246	904	178	10.5	4	1.8	40	3%
Determinate etiology													
23	44	M	White	S	14.3	8,052	10153	219	2.6	2	3.9	40	75%
24	52	M	White	LT	12.2	2,581	6,259	183	5.6	1	1.0	37	15%
25	33	F	White	LT	26.2	366	562	107	5.1	3	0.6	37	24%
26	34	F	White	D	20.9	394	187	252	4.5	4	0.9	35	29%

ALFSG, survival rate estimated by the ALF Study Group; ALP, alkaline phosphatase; ALT, alanine aminotransferase; AST, aspartate aminotransferase; Coma, West Haven criteria classification; INR, international normalized ratio; MELD-Na, model for end-stage liver disease with serum sodium; TBil, total bilirubin.

S, LT, and D of the outcome represent spontaneous survival, liver transplant, and death, respectively; dash (−): missing data.

Patients 23–26 were previously considered to have IND-ALF, but their etiologies were recently identified by the ALFSG. Patient 23 was an acetaminophen hepatotoxicity case; the condition of patient 24 was due to niacin and rosuvastatin; the condition of patient 25 was induced by hepatitis A infection; and the condition of patient 26 was associated with pregnancy.

Four patients with ALF were previously considered to have IND-ALF, but the etiologies were recently identified by ALFSG. Patient 23 was an acetaminophen hepatotoxicity case. The condition of patient 24 was due to niacin and rosuvastatin and that of patient 25 was induced by hepatitis A infection. The case of patient 26 was associated with pregnancy. These patients were excluded from our analysis but were used to validate the predictive model.

### WES analysis

The WES experimental process was described in detail previously (21). WES data of 22 patients were processed following the workflow of Genome Analysis Toolkit version 4 (GATK4) (47), and all parameters were set to default values. The individual sequence read was initially mapped to the Genome Reference Consortium Human Build 38 (GRCh38) (48). Duplicate reads were marked and sorted in a coordinate order, followed by base quality score recalibration for identifying the variant allele: SNP and insertion/deletion. Heterozygous genotypes with allele balance ≥0.9 and ≤0.2 were deemed homozygous alternative and reference alleles, respectively. The identified genetic variants were annotated by SnpEff (49), which predicts the impact and the transcript biotype of a variant. Variants were filtered by genotype quality ≥20, depth of coverage ≥10 (5 for sex chromosomes), putative impact greater than low, and the transcript biotype associated with protein coding. The qualified variants were selected for further analysis.

**Figure 4.**
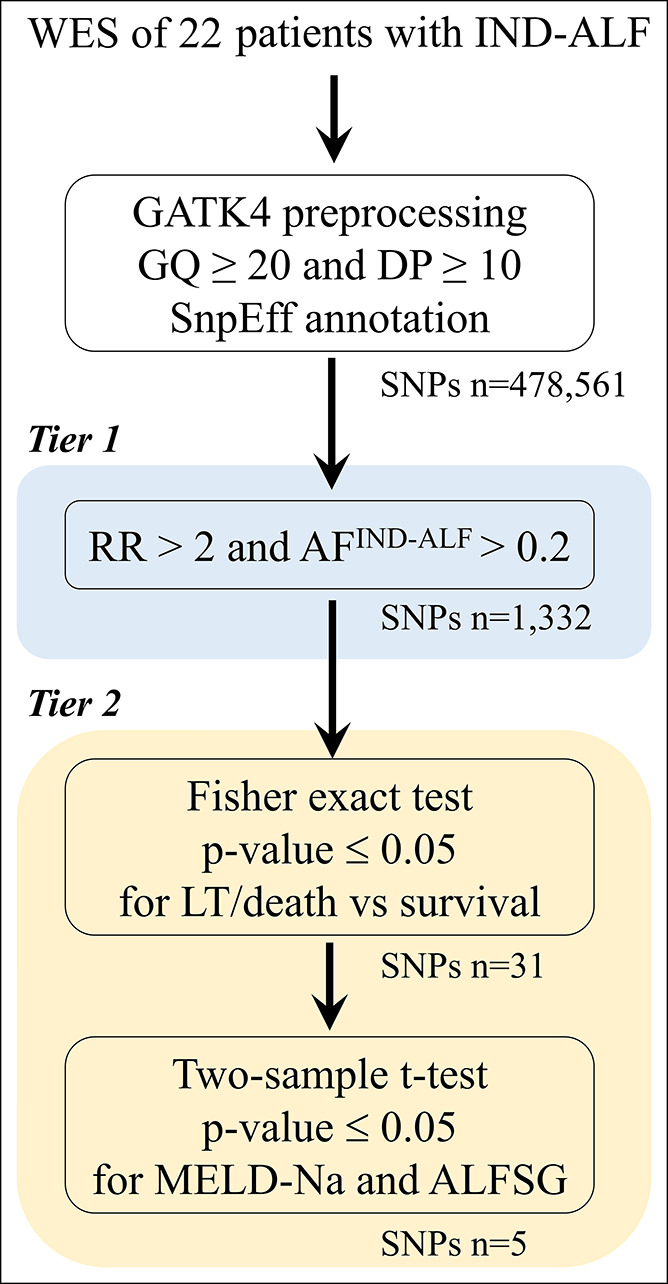
Procedure of WES data analysis for the 22 patients with indeterminate acute liver failure (IND-ALF). The sequence reads were preprocessed by using GATK4. The identified single-nucleotide polymorphisms (SNPs) were filtered by the genotype quality (GQ) ≥20, depth of coverage (DP) ≥10, and SnpEff-predicted properties, yielding 478,561 SNPs. A two-tier approach was used. In tier 1, 1,332 SNPs with relative risk (RR) > 2 and allele frequency of IND-ALF (AFIND-ALF) >0.2 were selected. In tier 2, the Fisher exact test was first applied for liver transplantation (LT)/death vs survival groups, and 31 SNPs with *P*-value ≤0.05 were retained. The 31 SNPs were then evaluated by the 2-sample *t* test, and only 5 SNPs were performed at *P*-value ≤0.05 for both MELD-Na scores and ALFSG indexes.

The genome aggregation database (gnomAD) provides the distribution of variant alleles in diverse human populations (50). We used allele frequencies in gnomAD version 3.1.1 as those of the control population. A 2-tier approach was used to identify the alleles associated with the prognosis of patients with IND-ALF (Figure 4). In tier 1, the RR and allele frequency were used together to identify the alleles with a relatively high frequency in the IND-ALF population. For the control group, an individual's allele count and allele number were extracted from non-Finnish European (n = 34,029), African American (n = 20,744), and Eastern Asian (n = 2,604) populations. The allele frequency of the control group was calculated by dividing the combined allele counts by the combined allele numbers. RR was evaluated by comparing the allele frequency in the study population (22 patients with IND-ALF) to that of the control group (gnomAD).

In tier 2, clinical outcomes (liver transplantation/death versus spontaneous recovery), MELD-Na scores, and ALFSG prognostic scores were used as surrogates of the phenotype to establish the association between the allele presence and the patient prognosis. The Fisher exact test was used to examine the association of the presence of an allele with the possibility of developing severe clinical outcomes, such as liver transplantation or death, and alleles with *P*-value ≤ 0.05 were considered statistically significant and were selected. Next, the Student *t* test was used to filter the selected alleles. For each allele, patients with this allele were assigned to group 1; others were placed in group 2. The MELD-Na score and ALFSG prognostic score were used to test the differences between groups 1 and 2. The 1-tailed *t* test with *P*-value ≤ 0.05 suggests that MELD-Na scores or ALFSG prognostic scores were significantly different among patients in groups 1 and 2.

### Statistical power

Statistical power is the probability of rejecting null hypothesis. The power level is determined by the odds and sample sizes between 2 groups (51). Sample sizes in groups A (patients with IND-ALF, n = 22) and B (general population, n = 577,377) are denoted by *n*_*A*_ and *n*_*B*_, and the probabilities of outcome are *p*_*A*_ and *p*_*B*_. The corresponding odds are calculated by $p_{A}\left(1-p_{A}\right)$ and $p_{B}\left(1-p_{B}\right)$. Statistical power is defined as follows:

$$1-\beta =\text{Φ}\left(\text{z}-z_{1-\frac{\alpha }{2}}\right)+\text{Φ}\left(-\text{z}-z_{1-\frac{\alpha }{2}}\right)$$

where α, β, and Φ represent the type I error, type II error, and standard normal distribution function, and z is defined as follows:

$$z=\frac{\ln\left(OR\right)}{\sqrt{\frac{1}{n_{A}p_{A}\left(1-p_{A}\right)}+\frac{1}{n_{B}p_{B}\left(1-p_{B}\right)}}}$$

where OR stands for the odds ratio, $\frac{p_{A}\left(1-p_{A}\right)}{p_{B}\left(1-p_{B}\right)}$.

### Linkage disequilibrium

Linkage disequilibrium calculates the correlation of alleles at different loci (52,53). Occurrence frequencies of 2 different alleles, M and N, were denoted by *P*_*M*_ and *P*_*N*_, and the concurrence frequency of both alleles M and N was denoted by *P*_*MN*_. The coefficient of linkage disequilibrium, *D*, was calculated as follows: *D = P*_*MN*_−*P*_*M*_*P*_*N*_. The correlation coefficient between alleles M and N is defined as follows:

$$r=\frac{D}{\sqrt{P_{M}\left(1-P_{M}\right)P_{N}\left(1-P_{N}\right)}}$$

where *r* represents correlation coefficient and ranges from 0 to 1. While *r* = 0 indicates that alleles M and N are independent, *r* = 1 suggests that 2 alleles have identical occurrence frequency, that is, *P*_*MN*_ *= P*_*M*_ *= P*_*N*_.

### Polygenic risk score

PRS estimates the risk of patients with IND-ALF based on the genetic variants (54). PRS is calculated by the summation of the product of allele count and logarithm of OR:

$$PRS=\overset{n}{\underset{i}{\sum }}x_{i}\beta_{i}=\overset{n}{\underset{i}{\sum }}x_{i}\log{\left(OR\right)}_{i}$$

where *x* and *β* represent allele counts and logarithm of odds ratios for the presence of an allele between liver transplant/death and spontaneous recovery groups, respectively, and index *i* represents the different generic variants. The PRS was normalized by scaling 0%–100%. A higher score suggests a higher risk of disease.

### Receiver operating characteristic curve

The receiver operating characteristic curve was used to evaluate the performance of score classifiers (55,56). With a different classification threshold, a ROC curve shows the true-positive rate against the false-positive rate. True-positive rate is defined as TP/(TP + FN) and false-positive rate is defined as FP/(FP + TN), where TP, TN, FP, and FN represent true-positive, true-negative, false-positive, and false-negative, respectively. Overall accuracy can be measured by AUC (57). The values of AUC range from 0.5 (random selection) to 1.0 (perfect prediction).

## CONFLICTS OF INTEREST

**Guarantor of the article:** Minjun Chen, PhD.

**Specific author contributions:** Conception: M.C. and J.R. Data collection: J.R. Data analysis and interpretation: T.J., B.P., H.H., P.H., J.R., D.G., W.L., J.R., and M.C. Manuscript draft: T.J. and M.C. Revision and review: M.C., J.R., T.J., B.P., H.H., P.H., J.R., D.G., and W.L.

**Financial support:** None to report.

**Potential competing interests:** None to report.

**Study Highlights**

**WHAT IS KNOWN:**

✓ Acute liver failure (ALF) is an uncommon clinical condition which can lead to rapid deterioration of liver function in patients without preexisting liver disease. ALF is fatal and characterized by a high mortality rate, and about 5% of the cases were considered indeterminate ALF (IND-ALF). We analyzed the whole exome sequencing data in 22 patients with IND-ALF and identified the significant genetic variants that might be associated with IND-ALF clinical outcomes.

**WHAT IS NEW HERE:**

✓ We found 31 genetic variants associated with a higher clinical risk in the IND-ALF patients, and 11 belong to the HLA class II genes. Five variants in HLA-DRB5, HLA-DQA1, and IDO1 were associated with a higher probability of transplant-free survival.

**TRANSLATIONAL IMPACT:**

✓ Genetic variants of HLA class II were found in IND-ALF patients. Certain genetic variants in HLA genes were associated with IND-ALF transplant-free survival; this observation may help to elucidate the mechanism of IND-ALF and assist in its diagnosis and management.

## Supplementary Material
