# Supplementary material for: Whole Exome Sequencing Reveals Genetic Variants in HLA Class II Genes Associated With Transplant-free Survival of Indeterminate Acute Liver Failure
Source: Clin Transl Gastroenterol. 2022 Jun 2;13(7):e00502. doi: 10.14309/ctg.0000000000000502 (PMC10476814; doi:10.14309/ctg.0000000000000502)
Supplement: Supplementary file 1 [file ct9-13-e00502-s001.docx]

**Supporting Information**

Title: Whole exome sequencing reveals genetic variants in HLA class II genes associated with transplant-free survival of indeterminate acute liver failure

Authors: Tsung-Jen Liao^1^, Bohu Pan^1^, Huixiao Hong^1^, Paul Hayashi^2^, Jody A. Rule^3^, Daniel Ganger^4^, William M. Lee^3^, Jorge Rakela^5†^, Minjun Chen^1†^

^1^Division of Bioinformatics and Biostatistics, U.S. Food and Drug Administration (FDA) National Center for Toxicological Research, Jefferson, AR, USA

^2^Division of Hepatology and Nutrition, Office of New Drugs, FDA Center for Drug Evaluation and Research, Silver Spring, MD, USA

^3^Division of Gastroenterology and Hepatology, University of Texas Southwestern, Dallas, TX, USA

^4^Division of Gastroenterology and Hepatology, Northwestern University, Chicago, IL, USA

^5^Division of Gastroenterology and Hepatology, Mayo Clinic, Phoenix, AZ, USA

**Disclaimer**: This article reflects the views of the authors and does not necessarily reflect those of the U.S. Food and Drug Administration. Any mention of commercial products is for clarification only and is not intended as approval, endorsement, or recommendation. **^†^Authors for correspondence:**

Minjun Chen, PhD

Division of Bioinformatics and Biostatistics

National Center for Toxicological Research

U.S. Food and Drug Administration

3900 NCTR RD, Jefferson, AR, 72079

Tel: +1(870)543-7057

E-mail: [Minjun.Chen@fda.hhs.gov](mailto:Minjun.Chen@fda.hhs.gov)

Jorge Rakela, MD

Division of Gastroenterology and Hepatology

Mayo Clinic Alix School of Medicine

Phoenix, AZ 85040

Tel: +1(480)301-8000

E-mail: [rakela.jorge@mayo.edu](mailto:rakela.jorge@mayo.edu)

**Table S1. Genetic variants with relative risk (RR>2) and allele frequency of the 22 IND-ALF patients (AF^IND-ALF^>0.5)**

| Gene | SNP ID | Position | Mutation | Consequence | AF^IND-ALF^ | RR |
| --- | --- | --- | --- | --- | --- | --- |
| HLA-related | | | | | | |
| HLA-A | rs1143146 | chr6:29942581 | C🡪G | L10V | 0.57 | 3.70 |
|  | rs9260123 | chr6:29942652 | C🡪G | Intron | 0.57 | 6.48 |
|  | rs9260127 | chr6:29942701 | G🡪C | Intron | 0.61 | 4.56 |
|  | rs9256980 | chr6:29942705 | G🡪A | Intron | 0.57 | 9.52 |
|  | rs199474436 | chr6:29942953 | T🡪A | N90K | 0.52 | 19.73 |
|  | rs9256982 | chr6:29943223 | T🡪G | Intron | 0.52 | 2.05 |
|  | rs1136695 | chr6:29943287 | A🡪G | I121M | 0.64 | 4.21 |
|  | rs3173420 | chr6:29943337 | G🡪A | R138Q | 0.61 | 6.22 |
|  | rs3173419 | chr6:29943342 | G🡪T | D140Y | 0.55 | 42.32 |
|  | rs1059509 | chr6:29943377 | C🡪A | N151K | 0.57 | 13.35 |
|  | rs2735111 | chr6:29943761 | G🡪A | Intron | 0.57 | 9.17 |
|  | rs1136903 | chr6:29944503 | T🡪C | L300P | 0.66 | 2.52 |
|  | rs1141891 | chr6:29944591 | C🡪T | Synonymous | 0.66 | 4.11 |
|  | rs1655894 | chr6:29944716 | G🡪A | Intron | 0.70 | 2.35 |
|  | rs9260201 | chr6:29944891 | T🡪G | Intron | 0.66 | 4.88 |
|  | rs1137296 | chr6:29945075 | T🡪C | Synonymous | 0.64 | 5.20 |
|  | rs2231119 | chr6:29945079 | A🡪T | T345S | 0.64 | 5.41 |
| HLA-B | rs1050344 | chr6:31270457 | G🡪A | Synonymous | 0.64 | 46.75 |
|  | rs2308604 | chr6:31271074 | T🡪C | Synonymous | 0.64 | 8.56 |
|  | rs5006140 | chr6:31356468 | A🡪C | Intron | 0.57 | 2.48 |
|  | rs9266196 | chr6:31357052 | G🡪C | Intron | 0.55 | 9.33 |
|  | rs9266197 | chr6:31357053 | G🡪A | Intron | 0.57 | 9.96 |
|  | rs1050458 | chr6:31357148 | A🡪G | M4T | 0.57 | 4.36 |
|  | rs9266206 | chr6:31357154 | A🡪C | L2R | 0.57 | 3.95 |
|  | rs9266207 | chr6:31357176 | C🡪T | 5’ UTR | 0.55 | 3.42 |
| HLA-C | rs9264594 | chr6:31269271 | A🡪G | Intron | 0.64 | 2.16 |
|  | rs2894204 | chr6:31269284 | C🡪T | Intron | 0.64 | 3.09 |
|  | rs1130838 | chr6:31269347 | T🡪C | T363A | 0.64 | 6.43 |
|  | rs35708511 | chr6:31269385 | C🡪G | C350S | 0.64 | 10.19 |
|  | rs9264596 | chr6:31269453 | A🡪G | Intron | 0.64 | 32.64 |
|  | rs9264597 | chr6:31269456 | A🡪G | Intron | 0.64 | 40.51 |
|  | rs2523609 | chr6:31269478 | G🡪A | Intron | 0.61 | 77.16 |
|  | rs68094471 | chr6:31269546 | A🡪G | Intron | 0.64 | 138.72 |
|  | rs66772001 | chr6:31269556 | T🡪A | Intron | 0.64 | 187.52 |
|  | rs67827555 | chr6:31269576 | T🡪C | Intron | 0.64 | 321.30 |
|  | rs56010430 | chr6:31269628 | C🡪T | Intron | 0.64 | 509.21 |
|  | rs66459704 | chr6:31269660 | T🡪C | Intron | 0.64 | 952.21 |
|  | rs68037221 | chr6:31269672 | T🡪C | Intron | 0.64 | 11.054 |
|  | rs66620546 | chr6:31269686 | C🡪G | Intron | 0.59 | 822.18 |
|  | rs9264608 | chr6:31269883 | A🡪G | Intron | 0.64 | 5773.86 |
|  | rs9264609 | chr6:31269887 | C🡪T | Intron | 0.64 | 3261.30 |
|  | rs9264610 | chr6:31269946 | G🡪A | Intron | 0.64 | 2608.28 |
|  | rs2074497 | chr6:31269950 | T🡪C | Intron | 0.64 | 3166.55 |
|  | rs1130935 | chr6:31269990 | T🡪C | M331V | 0.64 | 4768.06 |
|  | rs1050105 | chr6:31269992 | G🡪A | A330V | 0.64 | 5865.75 |
|  | rs1050106 | chr6:31269994 | G🡪A | Synonymous | 0.64 | 4091.46 |
|  | rs1130947 | chr6:31269996 | T🡪C | T329A | 0.64 | 5720.66 |
|  | rs41540512 | chr6:31269997 | G🡪C | Synonymous | 0.64 | 7301.64 |
| HLA-C | rs41556617 | chr6:31270009 | A🡪T | Synonymous | 0.64 | 4112.73 |
|  | rs1050147 | chr6:31270025 | A🡪G | V319A | 0.64 | 4842.31 |
|  | rs1050180 | chr6:31270056 | T🡪C | M309V | 0.64 | 4136.91 |
|  | rs9264622 | chr6:31270099 | A🡪G | Intron | 0.64 | 2194.09 |
|  | rs67298404 | chr6:31270154 | CAGA🡪C | Intron | 0.64 | 30661.29 |
|  | rs9264623 | chr6:31270173 | T🡪C | Intron | 0.61 | 204.74 |
|  | rs9264624 | chr6:31270178 | T🡪G | Intron | 0.61 | 220.09 |
|  | rs9264625 | chr6:31270183 | G🡪C | Intron | 0.59 | 217.90 |
|  | rs9264626 | chr6:31270185 | T🡪C | Intron | 0.59 | 215.66 |
|  | rs9264627 | chr6:31270186 | G🡪A | Intron | 0.59 | 216.27 |
|  | rs9264628 | chr6:31270194 | C🡪T | Intron | 0.59 | 203.85 |
|  | rs9264629 | chr6:31270198 | T🡪C | Intron | 0.59 | 183.78 |
|  | rs2308628 | chr6:31270214 | G🡪T | S297R | 0.59 | 151.92 |
|  | rs1131015 | chr6:31270233 | T🡪G | Q291P | 0.55 | 5017.97 |
|  | rs2308622 | chr6:31270252 | T🡪C | M285V | 0.59 | 199.12 |
|  | rs1050317 | chr6:31270358 | G🡪A | Synonymous | 0.64 | 21.85 |
|  | rs1050320 | chr6:31270361 | C🡪T | Synonymous | 0.64 | 21.87 |
|  | rs1050716 | chr6:31270453 | G🡪C | L218V | 0.64 | 42.69 |
|  | rs1131096 | chr6:31270482 | G🡪T | P208H | 0.64 | 621.85 |
|  | rs9264636 | chr6:31270520 | C🡪T | Intron | 0.64 | 51.78 |
|  | rs9264637 | chr6:31270531 | G🡪C | Intron | 0.64 | 50.74 |
|  | rs9264638 | chr6:31270541 | G🡪A | Intron | 0.64 | 451.50 |
|  | rs9264640 | chr6:31270595 | C🡪G | Intron | 0.64 | 30.08 |
|  | rs9264642 | chr6:31270713 | T🡪C | Intron | 0.57 | 24.29 |
|  | rs9264643 | chr6:31270715 | C🡪A | Intron | 0.64 | 17.83 |
|  | rs9264645 | chr6:31270747 | G🡪A | Intron | 0.64 | 56.58 |
|  | rs9264646 | chr6:31270758 | T🡪C | Intron | 0.64 | 110.32 |
|  | rs9264647 | chr6:31270836 | A🡪G | Intron | 0.61 | 93.33 |
|  | rs9264651 | chr6:31270951 | G🡪C | Intron | 0.64 | 66.27 |
|  | rs17884428 | chr6:31270982 | C🡪T | Intron | 0.55 | 39.81 |
|  | rs9264652 | chr6:31270985 | T🡪C | Intron | 0.64 | 40.72 |
|  | rs9264653 | chr6:31271037 | T🡪C | Intron | 0.52 | 40.37 |
|  | rs1050366 | chr6:31271180 | A🡪C | L171W | 0.59 | 8.43 |
|  | rs9264663 | chr6:31271428 | C🡪T | Intron | 0.64 | 9.29 |
|  | rs9264664 | chr6:31271450 | C🡪T | Intron | 0.64 | 7.00 |
|  | rs66565287 | chr6:31271493 | GATCCA🡪G | Intron | 0.64 | 4.07 |
|  | rs9264665 | chr6:31271502 | T🡪C | Intron | 0.64 | 3.80 |
|  | rs9264668 | chr6:31271845 | C🡪A | D33Y | 0.57 | 6.26 |
|  | rs9264669 | chr6:31271904 | A🡪T | Intron | 0.57 | 4.01 |
| HLA-DQA1 | rs3187964 | chr6:32637430 | C🡪G | 5’ UTR | 0.57 | 2.49 |
|  | rs1047989 | chr6:32637480 | C🡪A | L8M | 0.55 | 4.76 |
|  | rs9272441 | chr6:32637603 | T🡪G | Intron | 0.55 | 4.27 |
|  | rs9272442 | chr6:32637623 | C🡪T | Intron | 0.55 | 4.51 |
|  | rs707950 | chr6:32642096 | G🡪C | Q152H | 0.61 | 2.56 |
|  | rs9272756 | chr6:32642270 | T🡪C | Intron | 0.57 | 3.44 |
|  | rs34843907 | chr6:32642282 | T🡪G | Intron | 0.57 | 4.48 |
|  | rs9272858 | chr6:32642941 | C🡪T | Intron | 0.55 | 2.50 |
| HLA-DRB1 | rs9281872 | chr6:32584091 | G🡪GC | Intron | 0.52 | 4.83 |
| *Others* | | | | | | |
| CDK11A | rs77869096 | chr1:1713611 | C🡪T | Intron | 0.73 | 3.12 |
| CDK11A | rs33938712 | chr1:1714399 | CA🡪C | Intron | 0.80 | 8.44 |
| CDK11A | rs61777494 | chr1:1722599 | G🡪A | Intron | 0.77 | 2.32 |
| ANKRD36 | rs33971912 | chr2:97151827 | C🡪CT | Intron | 1.00 | 2.00 |
| FRMD1 | rs71004185 | chr6:168075852 | CGTGTCCACATTTCCGGTGCCCGGTGTCCACATTTCCGGCGTCCCGTGTCCACATTTCCGGTGCCCG🡪C | Intron | 0.75 | 40.93 |
| GTF2IRD2 | rs142434912 | chr7:74797024 | G🡪T | Q830K | 1.00 | 2.45 |
| RSPH10B | rs369329751 | chr7:5966800 | G🡪A | Intron | 0.70 | 47.48 |
| DENND2B | - | chr11:8718092 | A🡪AG | - | 0.55 | 14.55 |
| FAIM2 | rs1248263 | chr12:49877589 | G🡪T | Intron | 0.66 | 2.27 |
| P2RX2 | rs55726692 | chr12:132620863 | AGCCTGGGACTGACCCGGGCTCTCGAGGGGCCTCTCGTGTGCCCTTGTGACCCCCTTCCCTG🡪A | Intron | 0.70 | 3.12 |
| TCF25 | rs143440936 | chr16:89907369 | CTCCCAGT🡪C | Intron | 0.61 | 2.69 |
| ZNF28 | rs9917046 | chr19:52799646 | G🡪C | Non-coding | 0.86 | 8.84 |
| ZNF28 | rs9917044 | chr19:52799689 | C🡪T | Stop lost | 0.86 | 14.27 |


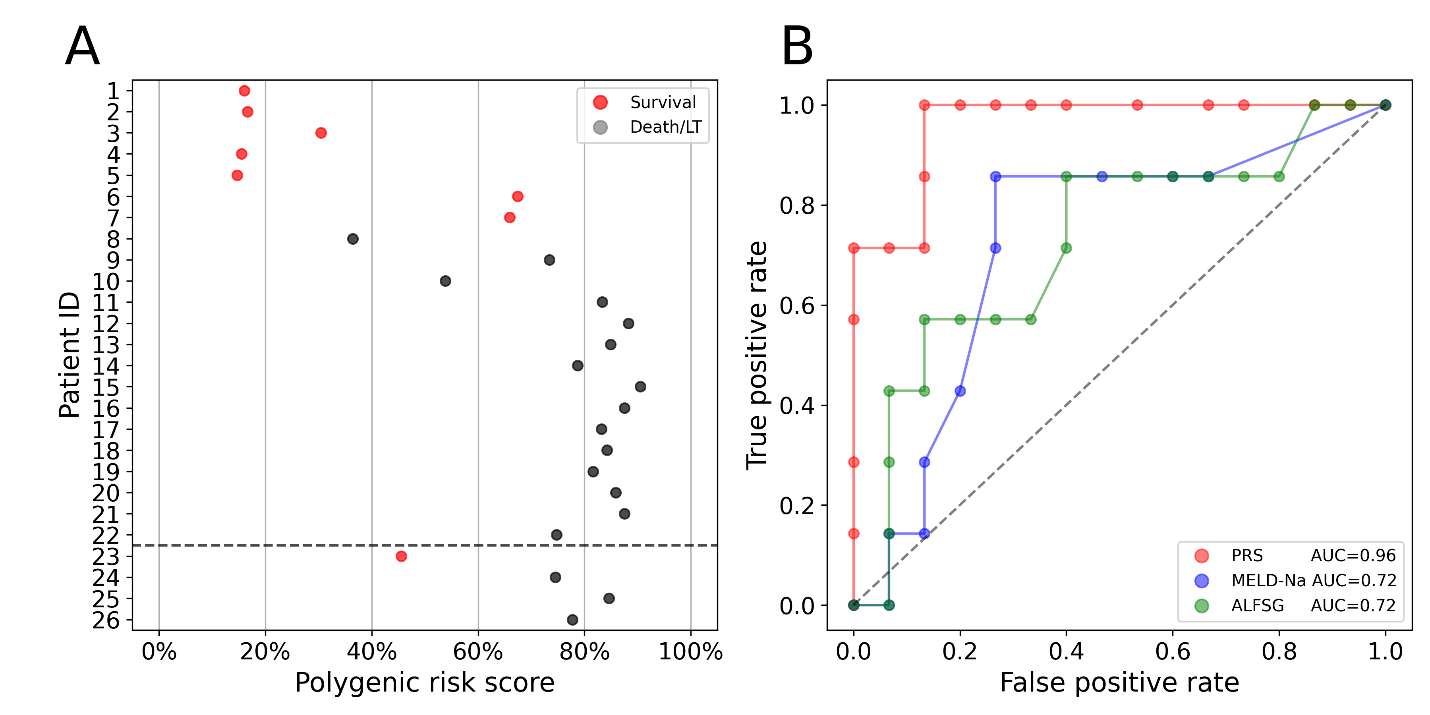


**Fig. S1. The polygenic risk score (PRS) using 31 SNPs from table 2 predicts the risk for the 22 IND-ALF patients and compares them to the MELD-Na scores and ALFSG indexes.** (A) The predicted risk scaled from 0% (lowest risk) to 100% (highest risk). The patients who survived spontaneously were colored red, and patients who died or underwent LT were colored grey. The dashed line separates the IND-ALF patients (1–22) and patients with identified etiologies (23–26). (B) The receiver operating characteristic (ROC) curve shows the true positive rate (TPR) against the false positive rate (FPR) at different classification thresholds. The areas under the curve (AUC) of PRS (red), MELD-Na (blue), and ALFSG (green) are 0.96, 0.72, and 0.72, respectively.


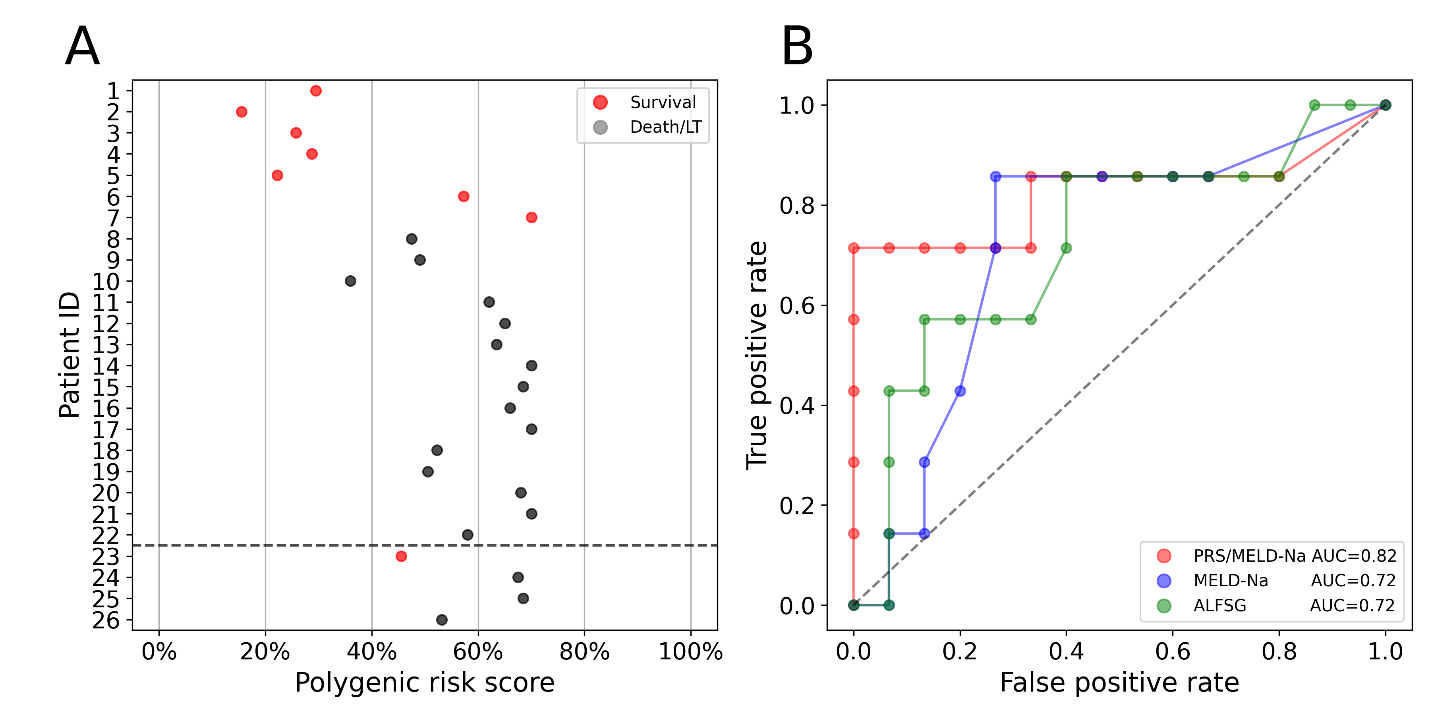


**Fig. S2. The combination of polygenic risk score (PRS) with MELD-Na predicts the risk for the 22 IND-ALF patients and compares them to the mere MELD-Na scores and ALFSG indexes.** (A) The predicted risk scaled from 0% (lowest risk) to 100% (highest risk). The patients who survived spontaneously were colored red, and patients who died or underwent LT were colored grey. The dashed line separates the IND-ALF patients (1–22) and patients with identified etiologies (23–26). (B) The receiver operating characteristic (ROC) curve shows the true positive rate (TPR) against the false positive rate (FPR) at different classification thresholds. The areas under the curve (AUC) of PRS (red), MELD-Na (blue), and ALFSG (green) are 0.82, 0.72, and 0.72, respectively.


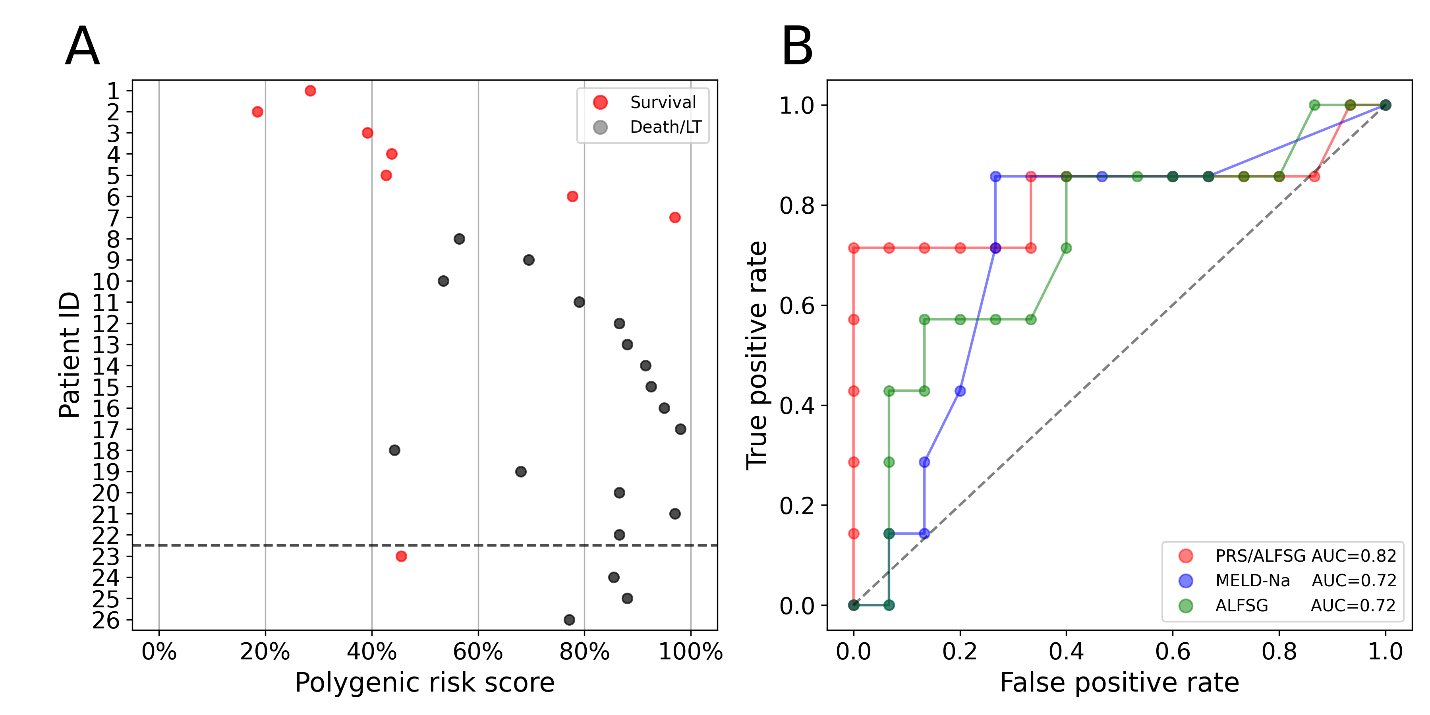


**Fig. S3. The combination of polygenic risk score (PRS) with ALFSG predicts the risk for the 22 IND-ALF patients and compares them to the MELD-Na scores and mere ALFSG indexes.** (A) The predicted risk scaled from 0% (lowest risk) to 100% (highest risk). The patients who survived spontaneously were colored red, and patients who died or underwent LT were colored grey. The dashed line separates the IND-ALF patients (1–22) and patients with identified etiologies (23–26). (B) The receiver operating characteristic (ROC) curve shows the true positive rate (TPR) against the false positive rate (FPR) at different classification thresholds. The areas under the curve (AUC) of PRS (red), MELD-Na (blue), and ALFSG (green) are 0.82, 0.72, and 0.72, respectively.


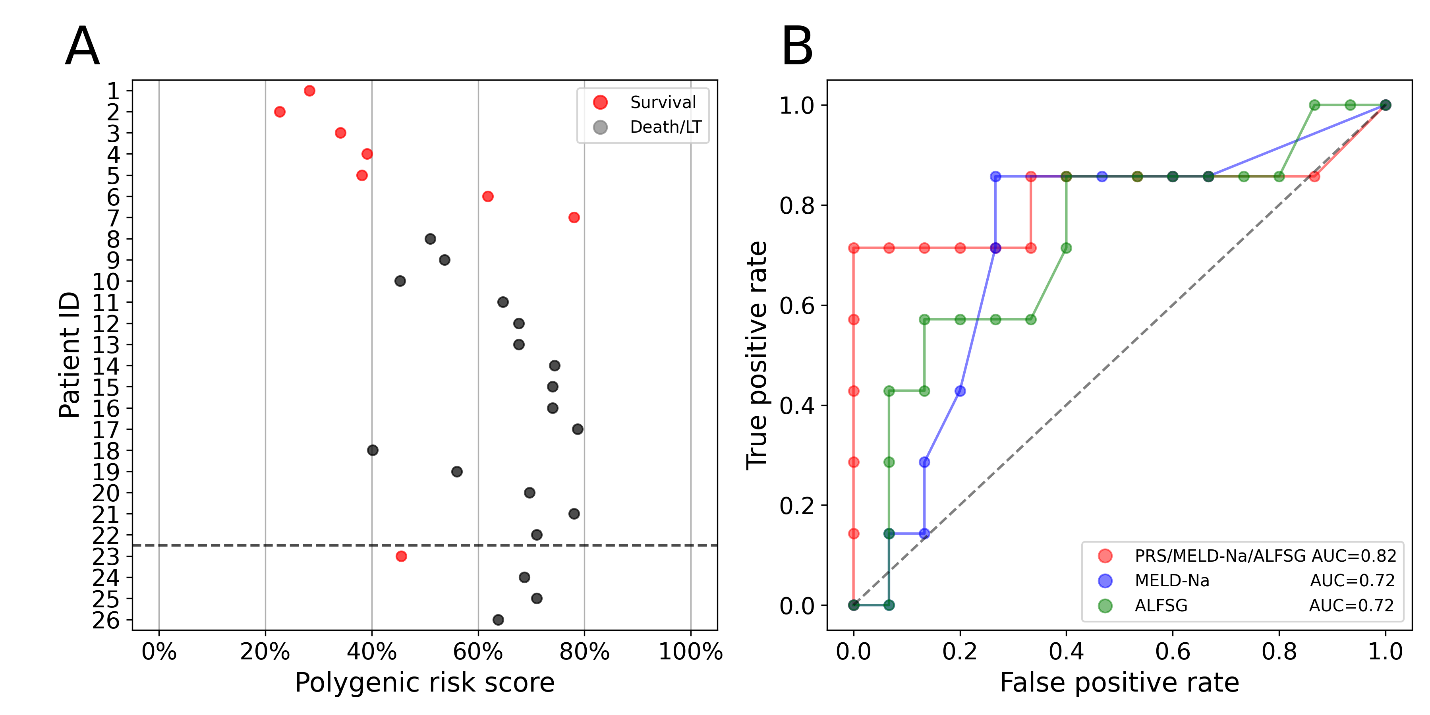


**Fig. S4. The combination of polygenic risk score (PRS) with MELD-Na and ALFSG predicts the risk for the 22 IND-ALF patients and compares them to the MELD-Na scores and ALFSG indexes.** (A) The predicted risk scaled from 0% (lowest risk) to 100% (highest risk). The patients who survived spontaneously were colored red, and patients who died or underwent LT were colored grey. The dashed line separates the IND-ALF patients (1–22) and patients with identified etiologies (23–26). (B) The receiver operating characteristic (ROC) curve shows the true positive rate (TPR) against the false positive rate (FPR) at different classification thresholds. The areas under the curve (AUC) of PRS (red), MELD-Na (blue), and ALFSG (green) are 0.82, 0.72, and 0.72, respectively.
